# In-vitro cytotoxicity of biosynthesized nanoceria using *Eucalyptus camaldulensis* leaves extract against MCF-7 breast cancer cell line

**DOI:** 10.1038/s41598-024-68272-3

**Published:** 2024-07-29

**Authors:** Fatemeh Abedi Tameh, Hamza Elsayed Ahmed Mohamed, Leila Aghababaee, Mahmood Akbari, Shervin Alikhah Asl, Mohammad Hasan Javadi, Marique Aucamp, Karen Jacqueline Cloete, Janet Soleimannejad, Malik Maaza

**Affiliations:** 1 UNESCO-UNISA-iTLABS Africa Chair in Nanoscience and Nanotechnology, College of Graduate Studies, University of South Africa, Muckleneuk Ridge, P.O. Box 392, Pretoria, 0003 South Africa; 2 School of Chemistry, College of Science, University of Tehran, P.O. Box 141556455, Tehran, Iran; 3 Neuroscience Laboratory, Institute of Biochemistry and Biophysics (IBB), Bio Organic, University of Tehran, Tehran, 1417614335 Iran; 4 Department of Chemistry, Sharif University of Technology, P.O. Box 11155‑9516, Tehran, Iran; 5 School of Pharmacy, University of the Western Cape, Robert Sobukwe Drive, Bellville, 7130 Cape Town South Africa

**Keywords:** Nanoceria, MCF-7 cell line, Breast cancer, MTT assay, Biosynthesis, *Eucalyptuscamaldulensis*, Biochemistry, Cancer, Chemical biology, Chemistry, Materials science, Nanoscience and technology

## Abstract

Cerium oxide nanoparticles possess unique properties that make them promising candidates in various fields, including cancer treatment. Among the proposed synthesis methods for CNPs, biosynthesis using natural extracts, offers an eco-friendly and convenient approach for producing CNPs, particularly for biomedical applications. In this study, a novel method of biosynthesis using the aqueous extract of *Eucalyptus camaldulensis* leaves was used to synthesize CNPs. Scanning electron microscopy and Transmission electron microscopy (TEM) techniques revealed that the synthesized CNPs exhibit a flower-like morphology. The particle size of CNPs obtained using Powder X-ray diffraction peaks and TEM as 13.43 and 39.25 nm. Energy-dispersive X-ray spectroscopy and Fourier transform infrared spectroscopy confirmed the effect of biomolecules during the synthesis process and the formation of CNPs. The cytotoxicity of biosynthesized samples was evaluated using the MTT method demonstrating the potential of these samples to inhibit MCF-7 cancerous cells. The viability of the MCF-7 cell line conducted by live/dead imaging assay confirmed the MTT cytotoxicity method and indicated their potential to inhibit cancerous cells. Furthermore, the successful uptake of CNPs by MCF-7 cancer cells, as demonstrated by confocal microscopy, provides evidence that the intracellular pathway contributes to the anticancer activity of the CNPs. In general, results indicate that the biosynthesized CNPs exhibit significant cytotoxicity against the MCF-7 cancerous cell line, attributed to their high surface area.

## Introduction

Cerium oxide nanoparticles (CNPs) have become one of the most promising nanomaterials, especially in medical and health fields in the last two decades^1^. CNPs have some unique physicochemical and biological therapeutic properties such as antibacterial, antiviral, antifungal^2^, anti-inflammatory^3^, anticancer^4^, antioxidant^5^, and multi-enzyme mimic activity^6^. Depending on surface characteristics and immediate environment, CNPs exert either anti- or pro-oxidative activity which regulates reactive oxygen species (ROS) levels in biological systems^7,8^. These applications are strongly dependent on the chemistry of cerium atoms and CNPs due to their screening effect and the nature of 4*f* orbitals. It has been reported that cerium can exist in both Ce^3+^ and Ce^4+^ oxidation states and due to the ease of switching between Ce^3+^ and Ce^4+^ at the nanoparticle surface, CNPs might have the potential of mimicking several natural enzymes, such as superoxide oxidase, catalase, oxidase, and phosphatase. Since these enzymes control intracellular ROS levels, CNPs may be able to prevent the growth of cancer^9–12^. Numerous researchers have made preliminary attempts at this technology. For instance, CNPs may cause oxidative stress and cytotoxicity in human lung cancer cells^13^.

The condition which is known as cancer is characterized by abnormal or uncontrolled cell growth that can spread to other healthy tissues of the body^14^. Breast cancer (BC), stands as the second major cause of cancer-related death among women worldwide. BC with over 2.3 million new cases, made about 11.7% of all cancer cases in 2023 Approximately 297,790 women and 2800 men were diagnosed with invasive breast cancer^15,16^. Fortunately, during the past 40 years, BC death and survival rates have decreased, with a 5-year relative survival rate above 90% in highly industrialized and highly developed countries^17,18^. Although modern treatment methods such as radiation, chemotherapy, and surgeries have shown to be successful, they have the potential of crippling adverse effects. Despite all the advanced treatments resulting in a 90% cure rate among patients who used the Chemotherapy strategy, the healthcare system still endures significant problems, including treatment resistance and malignancy recurrence^19,20^. Furthermore, since BC cells are highly heterogeneous majority of BC survivors have experienced a severe recurrence. Given these facts, current research is increasingly focused on developing more effective and less toxic treatment options for breast cancer^21,22^.

Several physical and chemical methods including precipitation^23^, hydrothermal^24^, solvothermal^25^, sol–gel^26^, emulsion method^27^, thermal decomposition^28^, electrochemical methods, microwave irradiation^29^, and laser ablation^30^, have been employed for synthesis of CNPs. The utilization of chemical or physical methods has led to environmental pollution due to the presence of toxic chemicals that are absorbed onto the surface of nanoparticles. These chemicals have detrimental impacts on medical applications, human health, and the environment. Consequently, in recent years, many eco-friendly methods called phytosynthesis or biosynthesis have been employed in the synthesis of CNPs using biological materials such as fungi, bacteria, and plant extracts. These methods are categorizing as Plant-Based Synthesis (which uses plant parts such as leaves, stems, flowers, bark, roots, fruits, vegetables, and shoots), amalgamation of nanoparticles using marine algae (which employs marine algae as reducing and capping agent), bacteria-mediated synthesis, fungi-mediated synthesis, actinomycetes-mediated synthesis, and yeast-mediated synthesis. These organisms play a crucial role in providing essential substances that can be utilized for the synthesis and manipulation of more organized nanoparticles. The method of obtaining nanoparticles through microbial mediation has proven to be significant, and plants can serve as a beneficial approach for nanoparticle formation. Plant extracts offer a convenient means of obtaining nanoparticles, as they have the ability to reduce metallic ions, facilitating the formation and stabilization of metallic nanoparticles by microbes. Additionally, plant extracts contain various components such as proteins, polysaccharides, amino acids, and phytochemicals like flavonoids, alkaloids, tannins, and polyphenols, which are emerging as potential stabilizers and reducers of nanoparticles^31^. These methods possess several advantageous qualities, making them highly desirable for various applications. Firstly, they are environmentally friendly, ensuring minimal harm to the ecosystem. Additionally, they are cost-effective, providing a cost-efficient solution without compromising on quality^32,33^. Furthermore, these methods are non-toxic and safe, ensuring the well-being of both users and the environment. Notably, they eliminate the need for special conditions, such as high pressure, energy, or temperature, commonly associated with chemical approaches. This eliminates the requirement for complex laboratory equipment and toxic precursor materials, enhancing convenience and accessibility^34,35^. These remarkable attributes make these methods particularly suitable for biomedical applications, offering a promising avenue for advancements in the field^36–39^.

In the past two decades, the use of plant extracts for the biosynthesis of CNPs has gained significant attention. One of the key benefits of using plant extracts is their renewable nature, as well as their mildness, non-toxic properties, and ability to act as stabilizing and reducing agents^32,40^. Plant extracts have emerged as an exciting resource for CNP synthesis, serving as both capping and reducing agents. Overall, using plant extracts in the biosynthesis of CNPs offers a promising and efficient approach. Utilizing plant extracts in the biosynthesis approach offers a safe and environmentally friendly alternative for synthesizing CNPs^32,41^. Recent studies have reported the successful synthesis of CNPs by natural reducing agents like plant extracts (such as *Ferula gummosa*^33^, *Aquilegia pubiflora*^38^, *Caccinia macranthera*^32^). These plant-based CNPs have demonstrated notable anticancer activities^42^. *Eucalyptus camaldulensis* (*E. camaldulensis*), a significant ethno-medicinal plant from the Myrtaceae family, has been traditionally used to treat sore throat and bacterial infections affecting the respiratory and urinary tracts^43^. The leaves of *E. camaldulensis* contain various phytochemical components, including cineol, cuminal, phellandrene, aromadendral, valeraldehyde, geraniol, cymene, catechol, tannins, terpenes, isoprenoids, phenolics, cardiac glycosides, sterols, saponins, and flavonoids. These compounds play a vital role in the formation of nanoparticles, making them vital components in the biosynthesis process^31,38^.

In this study, the leaf extract of *E. camaldulensis* was employed to facilitate the oxidation of cerium ions, present in the form of an aqueous solution of Ce(NO_3_)_3_, as flavonoids are believed to play a crucial role in the oxidation process for the biosynthesis of CNPs. The size, shape, morphology, and elemental composition of CNPs were analyzed using several techniques including Fourier transform infrared spectroscopy (FT-IR), Powder X-ray diffraction (PXRD), Scanning electron microscopy (SEM) coupled with energy-dispersive X-ray spectroscopy (EDX), Transmission electron microscopy (TEM), Diffuse reflectance (DR). Additionally, the cytotoxic effects of CNPs on MCF-7 human breast cancer were assessed using the MTT. Moreover, the viability of the MCF-7 cell line was conducted by live/dead imaging assay. Finally, to examine the cellular uptake and to determine if the cytotoxicity of the synthesized CNPs is influenced by the intracellular pathway, confocal microscopy was employed.

## Materials & methods

### Materials

Aqueous solutions were prepared using deionized water (DI). Analytical grade chemicals were procured that included cerium(III)nitrate hexahydrate (Ce(NO_3_)_3_·6H_2_O, 99%, Sigma-Aldrich), 3-aminopropyltriethoxysilane (APTES, NH_2_(CH_2_)3Si(OC_2_H_5_)_3_, 99%, Sigma-Aldrich), fluorescein isothiocyanate isomer I (FITC, C_21_H_11_NO_5_S, 90%, Sigma-Aldrich), ethanol (EtOH, Fluka 99.8%), acetone (Sigma-Aldrich 99.9%) and *N*,*N*-dimethylformamide (C_3_H_7_NO, DMF, Sigma-Aldrich), osmium tetroxide (OsO_4_, 99.98%, Sigma-Aldrich), Triton X-100 (Sigma-Aldrich), Dimethylformamide (DMF, 99.9%, Sigma-Aldrich), Dimethyl sulfoxide (DMSO, 99.9%, Sigma-Aldrich), Trypsinethylenediaminetetraacetic acid solution (EDTA, Biosera, France, 10×), Gibco RPMI-1640 and fetal bovine serum (FBS, Fisher Scientific U.S. Penicillin − streptomycin, 50×), 3-(4,5-dimethyl-2-thiazolyl)-2,5-diphenyl-2H-tetrazolium bromide (MTT, 98%, Sigma-Aldrich), 4′,6-Diamidino-2-phenylindole dihydrochloride, 2-(4-Amidinophenyl)-6-indolecarbamidine dihydrochloride (DAPI, 98%, Sigma-Aldrich).

### Preparation of aqueous extract of *E. camaldulensis* leaves

*Eucalyptus camaldulensis* leaves were obtained from iThemba Laboratory for Accelerator Based Sciences (Cape Town) South Africa (voucher number BOL0261549, Bolus herbarium, collected in June 2023), from its native habitat. The collected leaves were cleaned to remove dust and debris from the surface using a tap followed by DI, 3 times. For extraction, 100 g of leaves were placed in a 500 ml glass container filled with 200 ml DI. The solution was stirred and heated at 80 °C for 1 h then the solution was constantly stirred for 24 h in room temperature (25 °C). The resultant yellowish-brown leaf extract was cooled in a dark place for 3 h and then filtered using a paper filter to separate the residual material and stored in the refrigerator in a dark amber glass bottle to protect the leaf extract bioactive compounds from the effect of heat and light. The methods for the preparation of this plant extract for this study were carried out according to relevant guidelines and the previous report of Safdar et al.^44^.

### Synthesis of CNPs

**Figure 1:**
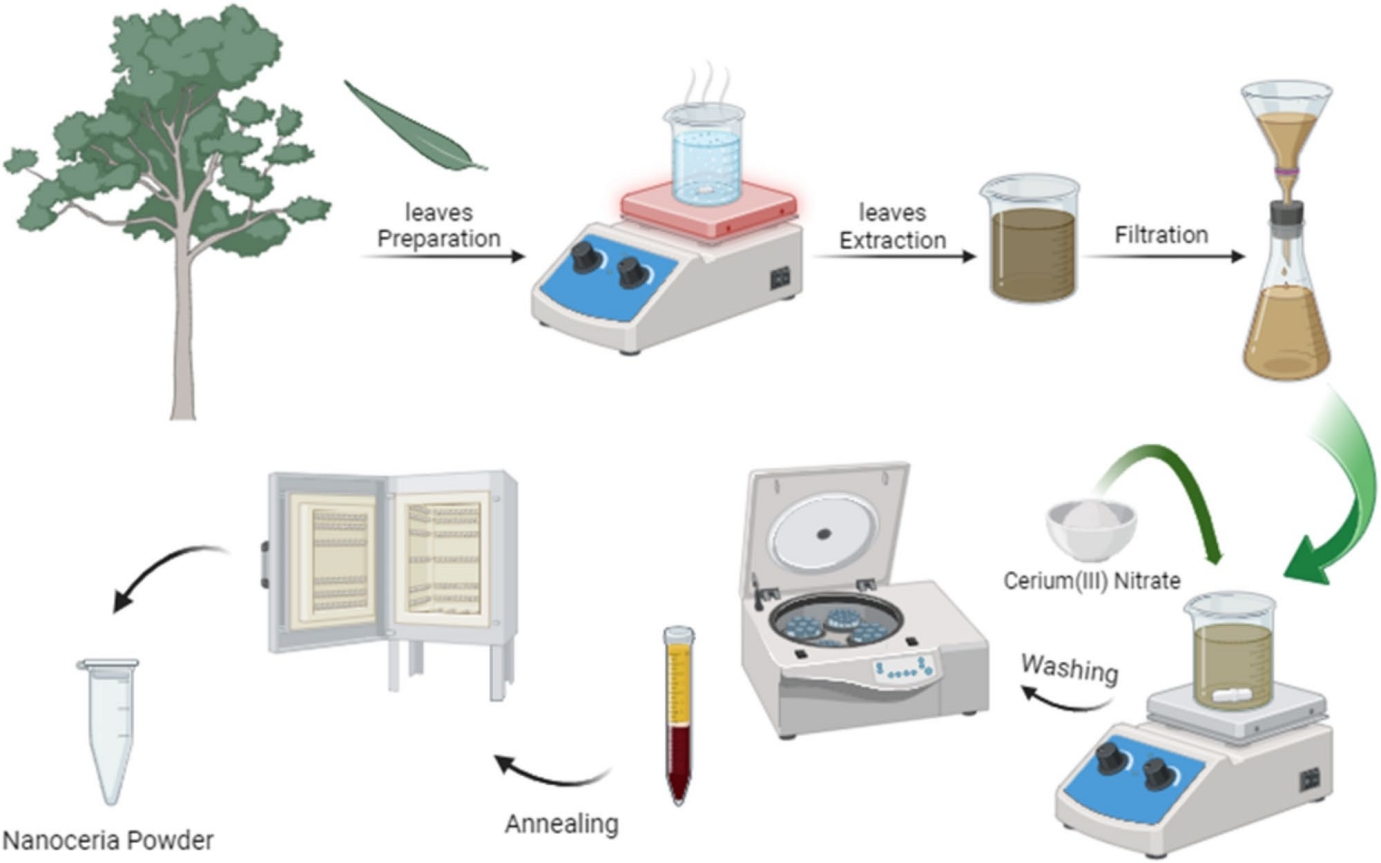
Schematic diagram of extraction and biosynthesis of CNPs.

CNPs were synthesized using aqueous extract of *E. camaldulensis* leaves (as an oxidizing and capping agent) by the biosynthesis method. 2g of Ce(NO_3_)_3_.6H_2_O powder was dissolved in 50 ml of prepared aqueous extract of *E. camaldulensis* leaves in a flask. Upon mixing and heating at 80 °C for an hour, and an aging process at room temperature for 24 h, the solution color changed from a yellowish-brown to a darker brown color indicating the formation of CNPs. Following an overnight (24 h) aging process, the solution was centrifuged and washed three times with DI and ethanol three times. This operation was done to get rid of any uncoordinated biomolecules and also to remove the excess cerium ions and prevent agglomeration. The dark brown precipitated CNPs were consequently dried in an oven at 60 °C for 4 h. The obtained powder was annealed at 400 °C for 2 h, resulting in a yellowish-white color powder of CNPs. The obtained powders were then stored in a vial for subsequent characterization and cytotoxicity and uptake testing. The detailed step-by-step process for preparing the extract and the biosynthesis of CNPs is shown in Fig. 1^44^. To ensure the repeatability of the biosynthesis process in this study, we repeated the experiment three times.

### Characterization of CNPs

In order to determine the functional groups of biosynthesized CNPs, FT-IR analysis was carried out within the wavenumber of 4000–600 cm^−1^. Diffuse reflectance (DR) spectroscopy, was employed to confirm the formation of CNPs in nano-sized and calculate the band gap energy using Origin 2022 software and Tauc plot. The crystalline structure and crystallite size were obtained using PXRD. PXRD patterns were recorded in the 2θ range of 5–80° using a Bruker D2 Phaser diffractometer. The device was operated following the Bragg–Brentano geometry using monochromatic Cu Kα radiation with a wavelength of 1.5406 Å, at a current of 30 mA and a voltage of 30 kV. The Debye–Scherrer equation, based on the width at half maximum, was utilized to estimate the crystallite size by HighScore Plus software. SEM and TEM techniques were employed to confirm the shape, morphology, and particle size of the CNPs. SEM characterization, combined with EDX analysis using a Tescan MIRA3 GMU Univac SEM equipped with a secondary electron detector and Drawbeam advanced/offline software, was utilized to verify the surface morphology and geometrical characteristics of the CNPs. To obtain a more detailed examination of the morphology, precise particle size, size distribution histogram, and TEM analysis were conducted. TEM observations were carried out using an FEI Tecnai 20 transmission electron microscope operating at 200 kV (Expander Lab6). The Image J software (version 1.53v) was used for accurate measurement of the TEM diameter.

### Functionalization of CNPs using fluorescein isothiocyanate (FITC)

In order to label biosynthesized CNPs to investigate their uptake and distribution profile in the MCF-7 cells, they were first involved in the initial functionalization using APTES. This step involved mixing 40 mg of sample 2 with 500 μl of APTES and 10 ml of ethanol at room temperature and stirring for 24 h. The resulting amino-functionalized CNPs were then washed with ethanol and centrifuged at 7000 rpm 3 times. The obtained precipitate was dried at room temperature for 24 h. An amount of 4 mg of the washed and dried amino-functionalized CNPs was suspended in 1 ml of a 1 mg/ml FITC solution in DMF. The suspension was stirred at room temperature for 4 h, washed once with acetone and twice with phosphate-buffered saline (PBS), followed by centrifugation at 7000 rpm. The resultant orange precipitate was diluted with PBS to achieve a concentration of 60 μg/ml, which was kept in a refrigerator for the uptake study^45^.

### Cell culture and MTT assay

The MCF-7 cell line was obtained from the National Cell Bank of Iran, Pasteur Institute of Iran. Cells were cultured in RPMI-1640 medium (Roswell Park Memorial Institute medium) supplemented with 10% heat inactivated fetal calf serum and streptomycin (100 µg/ml) and penicillin (100 IU/ml) and incubated at 37 °C in a humidified 5% CO_2_ atmosphere. All cells were subconfluently grown and passaged by treating them with 0.05% trypsin–EDTA.

For the MTT assay, approximately 1.0 × 10^4^ cells were seeded in pre-filled 96-well plates and incubated at 37 °C. When the cells reached approximately 80% confluency, they were either treated or left untreated (as control) for 24 h, 48 h, and 72 h with varying concentrations of 10, 20, 40, 80, and 100 µg/ml of the CNPs. As a reference for 100% toxicity, cell cultures treated with 4% Triton for 1 h were included. An MTT solution in PBS was added to each well, resulting in a final concentration of 500 μg/ml, and incubated at 37 °C for 4 h. After removing the supernatant carefully, the remaining formazan product was dissolved in DMSO, and the absorbance was measured at 492 nm using a multiwall scanning spectrophotometer (ELISA reader).

### Cellular uptake

MCF-7 cells were seeded in a 6-well plate at a density of 1 × 10^5^ cells per well and incubated with the growth medium at 37 °C for 24 h. The cells were then treated with either 60 μg/ml of FITC-labeled or non-labeled (as control) CNPs for 24 h. Afterward, the medium was removed, and the cells were washed immediately using PBS. The cells were then trypsinized using a trypsin/EDTA solution (2X) and centrifuged at 4000 rpm for 5 min. The resulting cell pellets were re-suspended in PBS, fixed with 1.5% PFA for 5 min, and washed with DI. The cell nuclei were stained with a concentration of 1 μM of DAPI for 15 min. Finally, the cells were washed with DI and prepared for imaging using a Nikon Eclipse Ti-E inverted confocal laser microscope (Japan)^46–48^.

### Live/dead cell imaging assay

To evaluate and validate the viability and cytotoxicity of CNPs, a Live/Dead assay cell imaging kit was employed in addition to the MTT assay results. This two-color assay enables the differentiation between live and dead cells using specific dyes. The non-fluorescent calcein AM dye stains live cells, undergoing conversion into a green-fluorescent dye that indicates intracellular esterase activity. Conversely, the cell-impermeable dye ethidium homodimer-1 stains dead and dying cells, resulting in enhanced red fluorescence due to binding to nucleic acids, indicating loss of plasma membrane integrity. The MCF-7 cell suspension was seeded in 6-well plates at a density of 10,000 cells per well, with 2 ml of supplemented DMEM, and incubated for 24 h at 37 °C under a 5%, CO_2_ atmosphere. Subsequently, the cells were treated whit 80 μg/ml of the CNPs for 24 h then Wash with serum-free buffer or medium to remove serum. MCF-7 cells were incubated with calcein AM and Ethidium Homodimer-1 (final concentration of 2μM and 4μM, respectively) for 5 min prior to fluorescence imaging. Finally, the cells were washed with PBS and prepared for imaging using a Nikon Eclipse Ti-E inverted confocal laser microscope (Japan)^44–46^. The green fluorescence was captured with an excitation (E_x_) wavelength of 495 nm and an emission (E_m_) wavelength of 515 nm, while the red fluorescence was captured with an E_x_/E_m_ of 495 nm/635 nm. Images of each well were recorded.

## Results

### FT-IR

**Figure 2:**
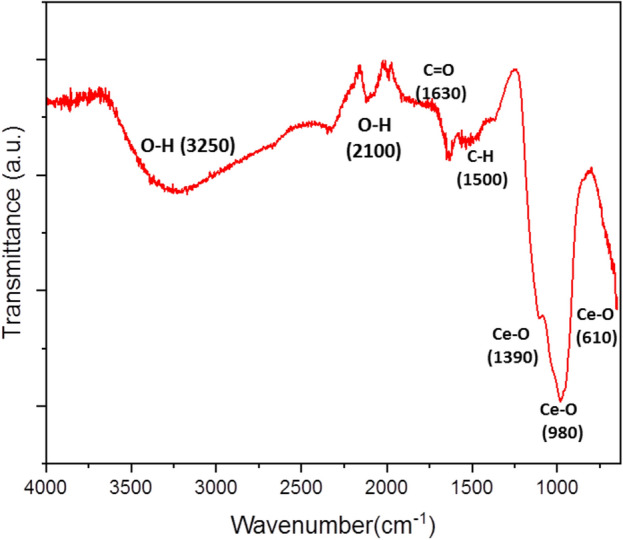
FT-IR spectra of CNPs in range of 4000–600 cm^-1^**.**

The FT-IR spectra of the obtained CNPs in the range of 4000–600 cm^−1^ are shown in Fig. 2. Observed board and strong bands at ~ 3250 cm^−1^ correspond to the hydroxyl (O–H) stretching of phenols and flavonoids in the reported *E. camaldulensis* extract FT-IR^31^. A prominent peak was observed at ~ 1630 and ~ 2100 cm^−1^ corresponding to the Carbonyl (C=O) and O–H stretch, indicating the presence of carboxylic acids and esters derived from phytochemical compounds present in the plant extract^49,50^. Additionally, the ~ 1500 cm^−1^ band is attributed to the peaks that appear as C-H deformation vibration in the IR spectrum of *E. camaldulensis* extract^31^. Overall, these results provide further confirmation of the significant role played by plant extract biomolecules in the synthesis process. Also, the peaks at ~ 980 cm^−1^, ~ 1050 cm^−1^, and ~ 1390 cm^−1^ are similar to the characterized vibrations of ceria commercial powders, and the peak at ~ 610 cm^−1^ is known as the vibration of the Ce–O bond. Moreover, there are no other peaks related to organic or inorganic functional groups, such as SO_4_^2−^, NO_3_^−^, and CO_3_^2−^ in the FT-IR spectra.

### DR

**Figure 3:**
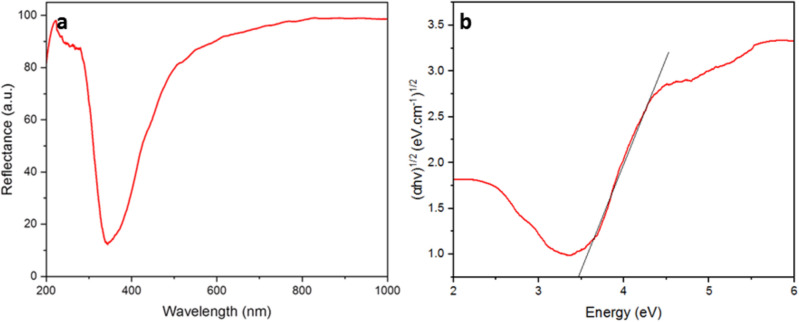
(**a**) DR spectrum of biosynthesized CNPs and; (**b**) Tauc plot based on DR of biosynthesized CNPs.

Reflectance spectra of the CNPs obtained using DR spectroscopy Fig. 3a For CNPs in the DR spectrum in the 350–400 nm wavelength range, a sharp band could be observed, signifying an absorption band attributed to the charge transfer from the 2*p* valence band of O^2−^ to the 4*f* band of Ce^4+^ as an indicator of the cerium oxide presence. This band is a typical characteristic of a sample of nano-sized cerium oxide, confirming the satisfying synthesis of CNPs^24^. Furthermore, DR spectrum confirms that synthesized CNPs are photocatalytic active^50^. The DR spectrum can be used to determine the optical band gap energy of CNPs. The optical band gap energy (Eg) can be computed using the absorbance spectrum of the powders, which Eg = 1240/*λ*_AE_, and *λ*_AE_ represents the edge absorbance wavelength. The optical band gap energy of biosynthesized CNPs using Tauc plot was calculated as 3.43 eV (Fig. 3b).

### PXRD

**Figure 4:**
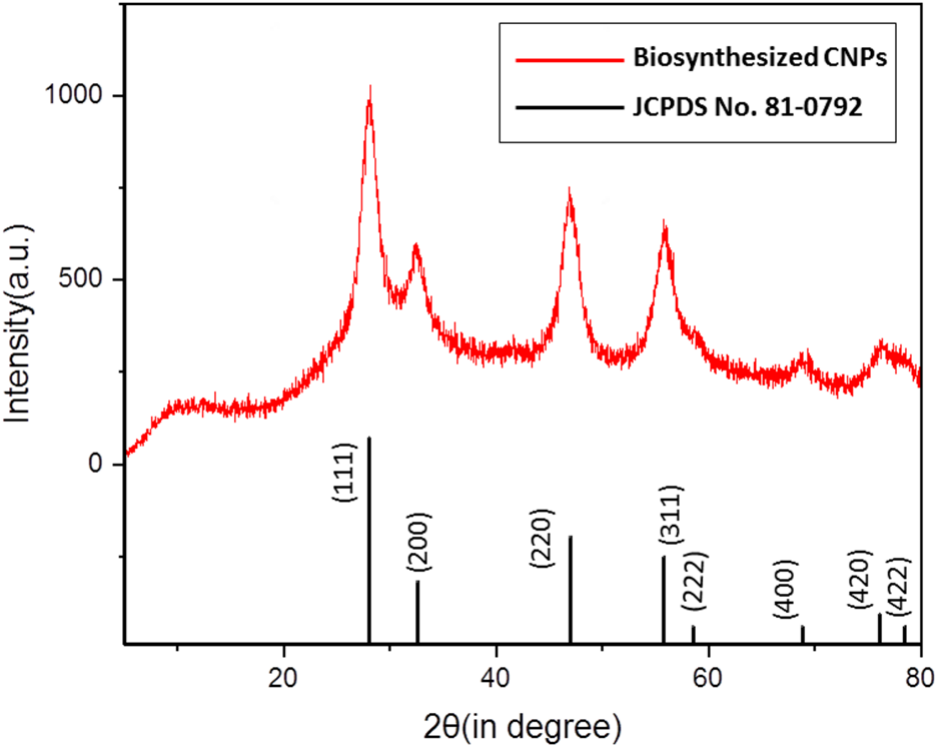
PXRD pattern of CNPs in the 2θ range of 5–80°.

The crystal structure and size of CNPs were determined through the PXRD characterization method (results are shown in Fig. 4). Diffraction pattern peaks corresponding to the respective crystal planes represent the cubic fluorite structure of CNPs having a space group of *Fm*3*m*(225) according to the JCPDS No. 81-0792 file. The results confirm the phase purity and the presence of CNPs crystal planes of (111), (220), (311), (200), (331), (222), (420), and (400) based on intensity. Furthermore, no peaks related to Ce(OH)_3_ with diffractions in (2θ = 23, 33, 48, 57, 60, 70, and 78 degrees) and/or Ce(OH)CO_3_ diffractions in (2θ = 25, 31, 44, 45, 48, 51, 54, and 58 degrees) have appeared which confirmed the CNPs as a pure product. The PXRD diffraction patterns of CNPs obtained in this study align with the findings of Ahmad et al., who also observed similar PXRD diffraction patterns when utilizing Phoenix dactylifera plant extract during the synthesis process of CNPs^48^. The average crystallite sizes using Debye–Scherrer’s equation via the Xpert HighScore software for the peaks of (111), (220), and (311) are calculated and obtained as 13.43 nm.

### SED & EDX

**Figure 5:**
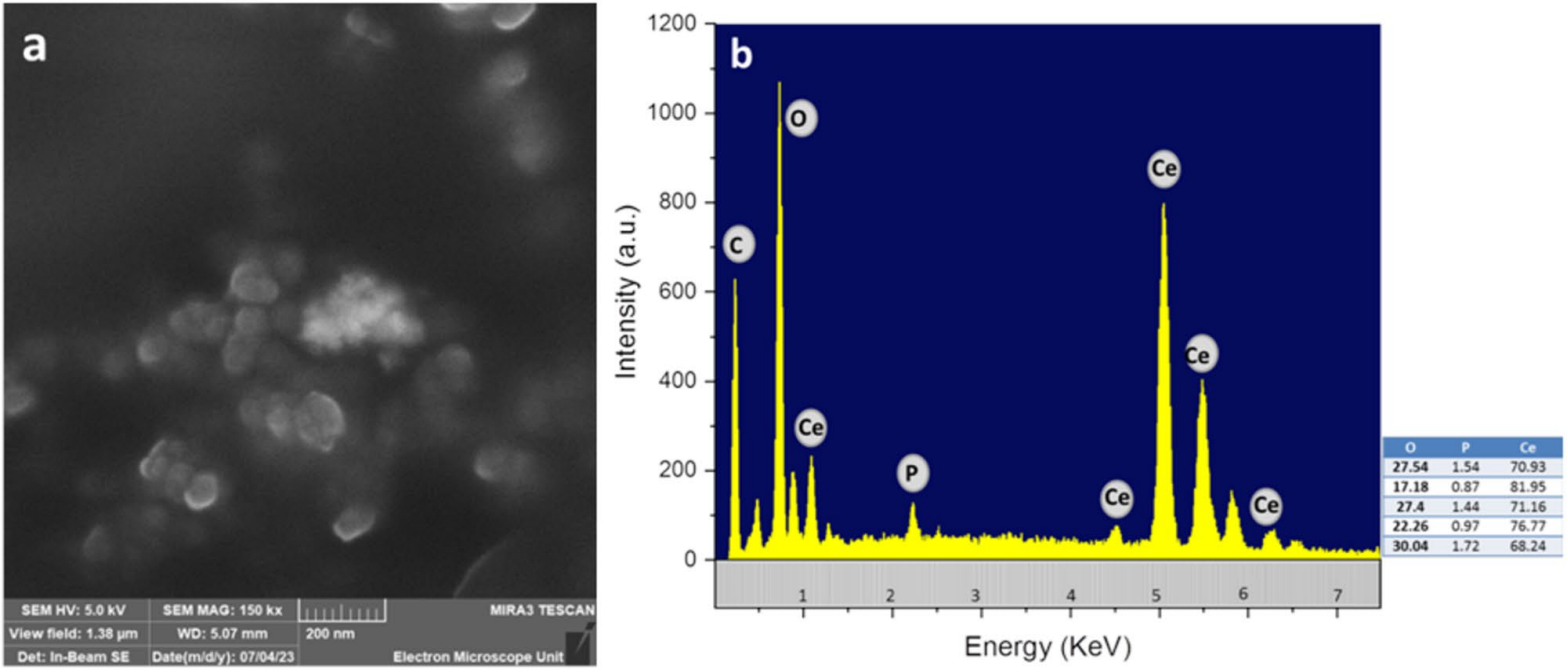
(**a**) SEM image and; (**b**) EDX spectra of CNPs.

SEM coupled with EDX analysis was conducted to investigate the elemental analysis, morphological characteristics, and structural arrangements of CNPs. The SEM images and EDX spectra of samples are shown in Fig. 5a,b. The size of CNP samples was smaller than 100 nm in all 3 dimensions. Furthermore, the morphology of the synthesized CNPs was flower-like and formed by aggregation of numerous very small sphere-like substructures (see Supplementary Figure S1). Reduction of particle size and the presence of biomolecules in *E. camaldulensis* leaves extract may support this observation. In addition, EDX was performed in order to characterize the elemental profile of the biosynthesized CNPs. The percentage of Ce and O and a small amount of phosphorus (P) were observed in the EDX data. Research shows that for the Ce atom there are strong peaks between 1.0 and 6.0 keV. The increase or decrease in the elemental composition of cerium is attributed to an oxidation state switching from + 3 to + 4 on the surface of CNPs. Additionally, the presence of peaks of carbon (C) and P with a very low percentage indicated the presence of biomolecules in *E. camaldulensis* extract. These characterizations and presence peaks for Ce and O confirm the formation and successful biosynthesis of CNP samples (see Supplementary Figure S2)^49–53^.

### TEM

**Figure 6:**
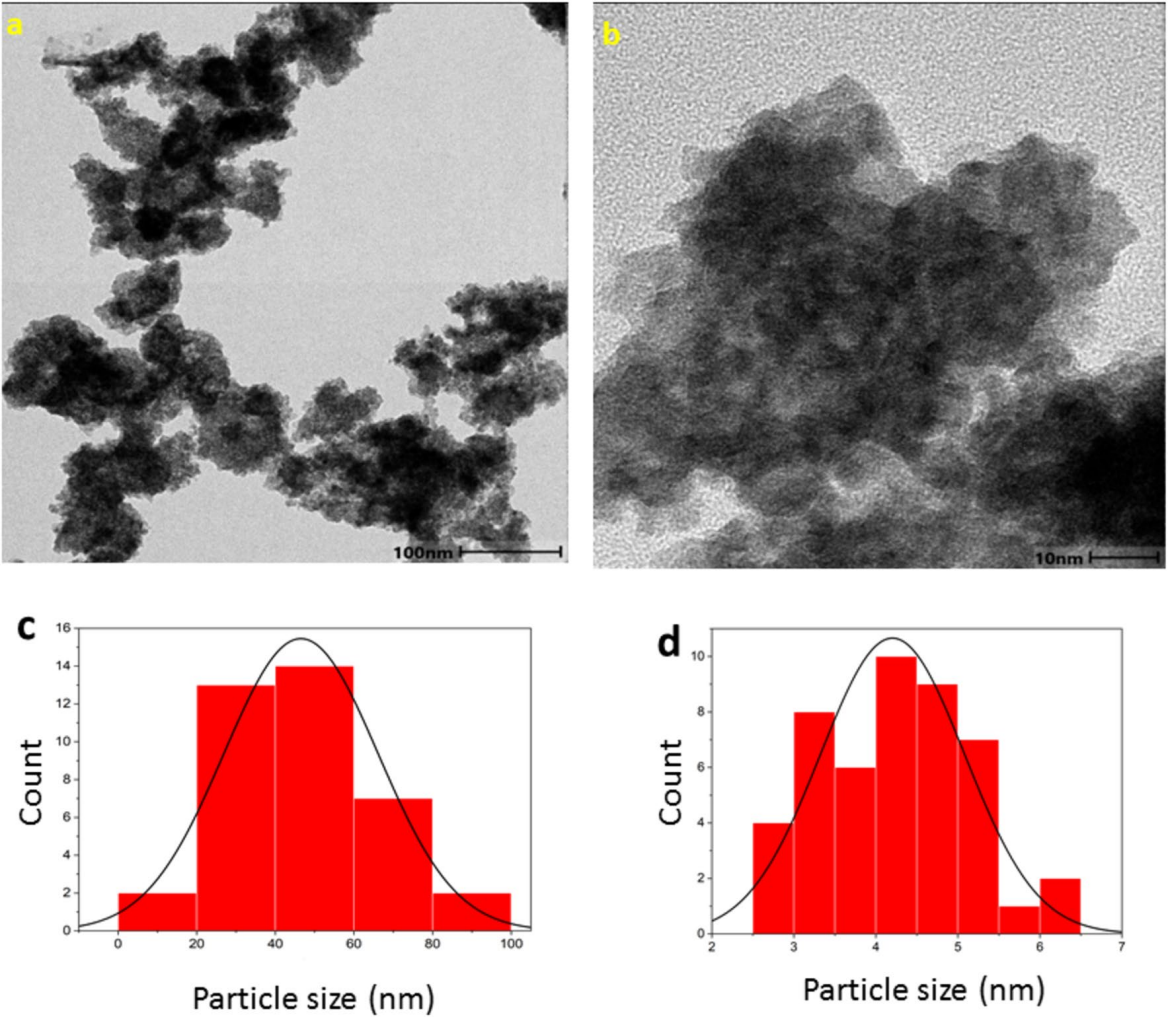
(**a**,**b**) TEM images of the CNPs at a resolution of 100 nm and 10 nm; (**c**) Histogram of flower-like structure of CNPs, (**d**) Histogram of sphere-like substructure of CNPs.

TEM analysis was performed to validate the SEM results and obtain accurate measurements of the size, morphology, and shapes of the CNPs. The obtained TEM images (Fig. 6a,b) confirmed the SEM results. The morphology seems to be flower-like composed of a large number of very small sphere-like CNPs. This morphology of the CNPs provides a significantly large surface area. It seems that the presence of biomolecules in the leaf extract is responsible for the synthesis of the nanomaterial in this particular shape. The size distribution of CNPs was in the range of 20–120 nm for flower-like structures and 2–10 nm for sphere-like substructures. The average size of flower-like structures was obtained as 39.25 nm (Fig. 6c) based on the histogram. Also histogram of size distribution for sphere-like substructures is indicated in Fig. 6d. The average size of the sphere-like substructures based on this histogram was calculated as 5.23 nm (see Supplementary Figures S3 and S4).

### Cytotoxicity test by MTT assay

**Figure 7:**
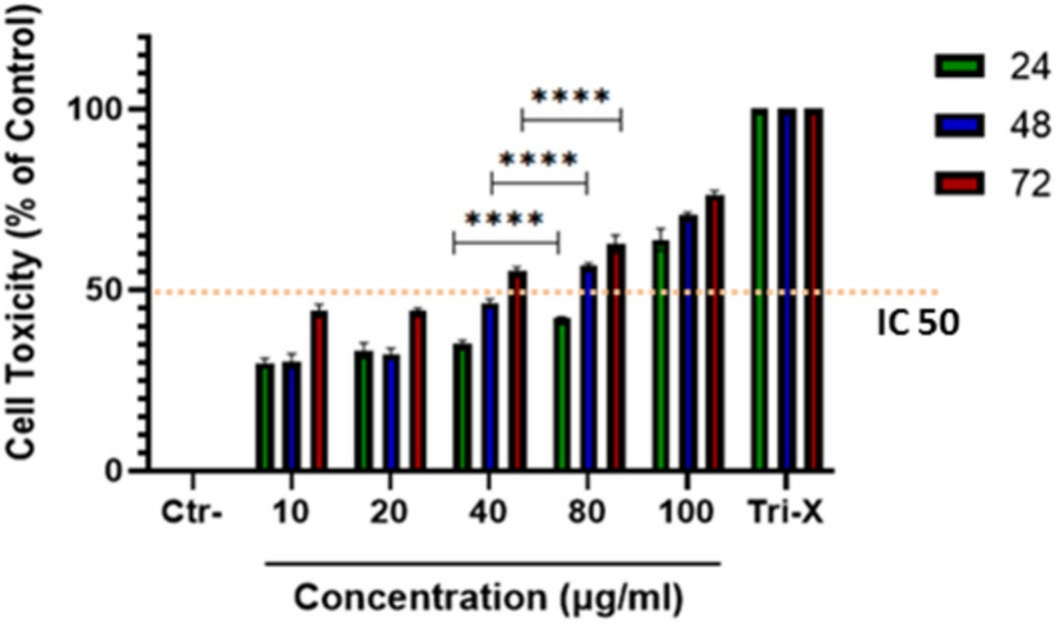
Cellular toxicity effect of CNPs against MCF-7 BC cell lines treated or not treated (Ctr) for 24 h, 48 h, and 72 h to different concentrations of: 10, 20, 40, 80, and 100 (µg/ml). The 1 h-treated cell cultures with 4% Triton were the reference of 100% toxicity (Tri-X). Data are represented as the mean ± SD. The test was done in triplicate (p-value ≤ 0.0001).

The results of cell viability and IC50 values are summarized in Fig. 7. As depicted in Fig. 7, cancerous MCF-7 cells exhibited a notable dose-dependent and time-dependent decrease in viability. The highest concentration of CNPs (100 μg/ml) reduced the viability of MCF-7 cells to 78%, 74%, and 69% after 24, 48, and 72 h respectively. Moreover, the concentration of 80 μg/ml showed comparable cytotoxicity within the same timeframe (43%, 58%, and 69%), indicating a significant increase in cytotoxicity over time. The concentration at which 50% (IC50) of cell death occurred at 40 µg/ml after 72 h, 80 µg/ml after 48 and 72 h, and 100 µg/ml after 24, 48, and 72 h. These findings provide evidence of substantial cytotoxicity and an inhibitory effect of biosynthesized CNPs against the MCF-7 cancerous cells, attributed to their small size, large surface area, and presence of biomolecules on their surface. In a previous study, Lin et al. reported viabilities of 88%, 78%, and 72% for CNPs with an average size of 10–100 nm after 24 h, 48 h, and 72 h, respectively, in the A549 lung cancer cell line^54^. In another study conducted by Panneerselvam et al. cytotoxicity of CNPs biosynthesized using *Echinochloa frumentaceae* grain extract was evaluated against MCF-7 cell line. This study demonstrated the presence of spherical CNPs with an average diameter ranging from 16 to 38 nm, exhibiting an IC50 value of 47.32 µg/ml^55^. In a study undertaken by Sridharan et al., the cytotoxicity of CNPs synthesized using an ethanolic extract of the *Brophyllam daigremontianum* plant was examined against the MCF-7 cell line. The findings revealed that the spherical nanoparticles displayed an IC50 value of 175.4 µg/ml^56^. In a study towards the anticancer activity of CNPs biosynthesized using Ananas Leaves Extract conducted by Alkhafagi et al. in this study, biosynthesized CNPs were coated by chitosan-linked folic acid to be delivered by Folate receptor positive cancer cells and evaluated the selective anticancer activity on MCF-7 breast cancer cell line. Obtained cytotoxicity in this study was assessed as 162.18 μg/ml in 48 h^57^. To make a comparison between the biosynthesized CNPs in this study with the performance of other nanoparticles for cancerous cells inhibition, we took a look at the of Hublikar et al. The biosynthesized silver nanoparticles in this study (using *Averrhoa bilimbi* leaf extract) exhibited an IC50 value of 49.52 μg/ml against the A549 cell line, while the IC50 for the MCF-7 cell line was 78.40 μg/ml^58^. The effect of the silver nanoparticles on both cancer cell lines was evaluated using the MTT assay, which demonstrated a dose-dependent cytotoxic effect. In another study, Silver nanoparticles were synthesized using ground nutshell and studied as an inhibitor for cancerous cells. The anti-cancer properties of these nanoparticles was evaluated employing MTT assay, and the IC50 value was found to be 80.25 μg/ml against A549 lung cancer cell line^59^. In a different study, *Pandanus amaryllifolius* Roxb leaf extract was utilized to synthesis silver nanoparticles. The cytotoxicity of the *Pandanus amaryllifolius* Roxb leaves-based silver nanoparticles was evaluated against both lung cancer (A549) and breast cancer (MCF-7) cell lines, and the results were compared with the standard cisplatin drug. The IC50 of these nanoparticles against MCF-7 and A549 cell lines was found to be 27.19 µg/ml and 22.36 µg/ml, respectively^60^.

The anticancer activity of CNPs has been established in previous studies and research work. However, the CNPs synthesized in this research not only employ a green and suitable method for biosynthesis, but also exhibit higher cellular toxicity at lower concentrations against MCF-7 cancerous cells compared to other nanoparticles used for anticancer applications (silver nanoparticles for instance). Moreover, the efficiency of this synthetic method, particularly in comparison to CNPs synthesized using other biosynthesis techniques, demonstrates that biosynthesized nanoparticles in this research are significantly more effective. This highlights the superior performance of the CNPs synthesized in the current study. It is speculated that this increased activity could be attributed to the higher surface area due to smaller size, specific morphology, higher prooxidant activity, and higher enzyme-mimic activity of the surface of biosynthesized CNPs. However, further investigations like extracellular enzyme-mimic tests are required to thoroughly examine and understand this phenomenon.

### Evaluation of CNPs uptake

#### Cellular uptake

**Figure 8:**
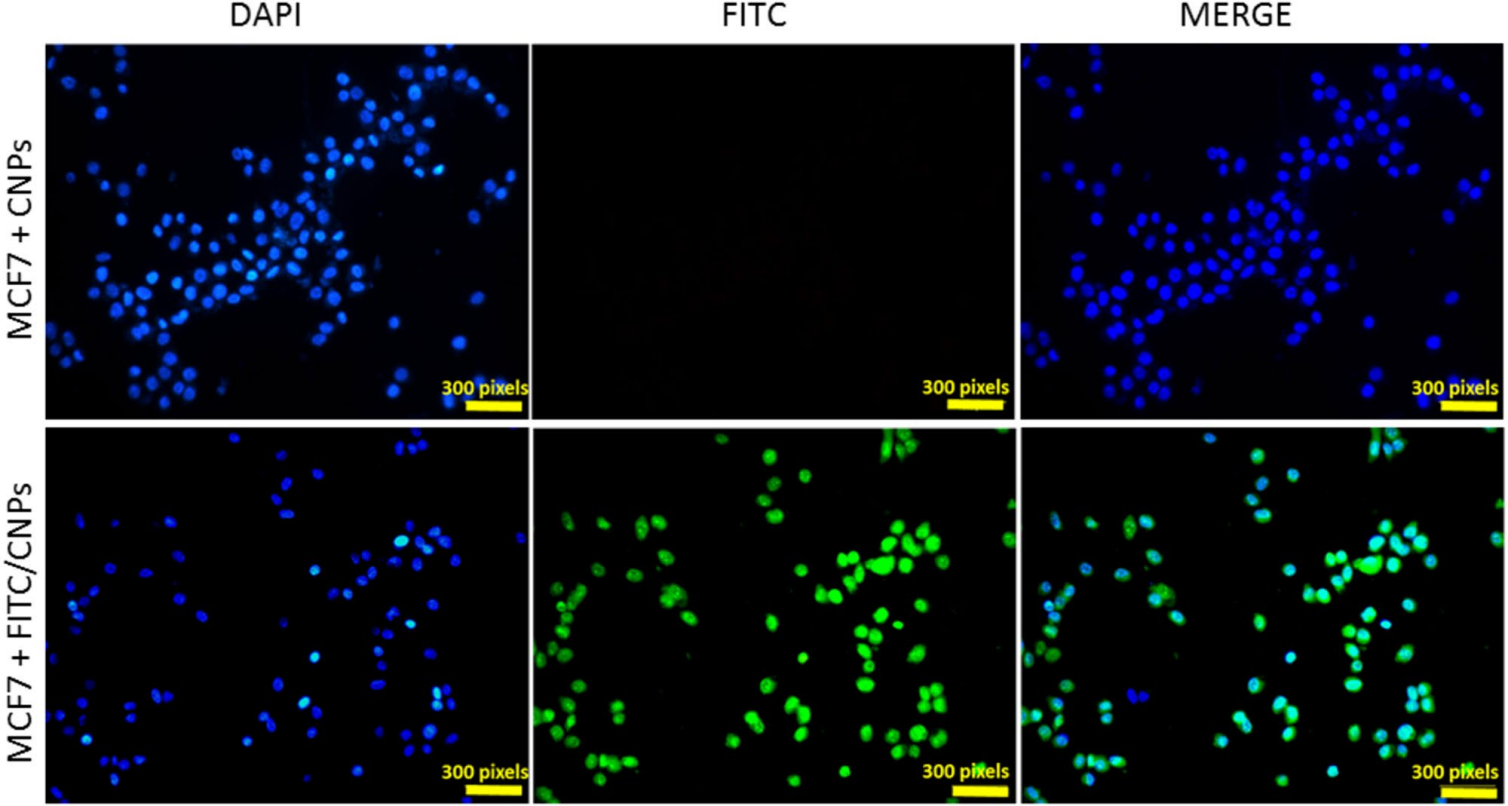
Cellular uptake; Confocal microscopic imaging of MCF-7 cells after incubation with 60 μg/ml of the FITC labeled CNPs and unlabeled CNPs for 24 h. The nuclei were stained with DAPI (blue).

Confocal microscopy was utilized to investigate cellular uptake and assess whether the intracellular pathway contributes to the cytotoxicity of the synthesized CNPs. Figure 8 presents a summary of the results, displaying confocal microscopic images of MCF-7 cells following a 24-h incubation with 60 μg/ml of both FITC-labeled and non-labeled CNPs. The images clearly demonstrate a substantial uptake of biosynthesized CNPs by MCF-7 cells. The confocal microscopic analysis confirms that the synthesized CNPs can penetrate the cell membrane of MCF-7 cells, suggesting that the inhibition of these cells may involve intracellular processes.

### Live/dead cell imaging assay

**Figure 9:**
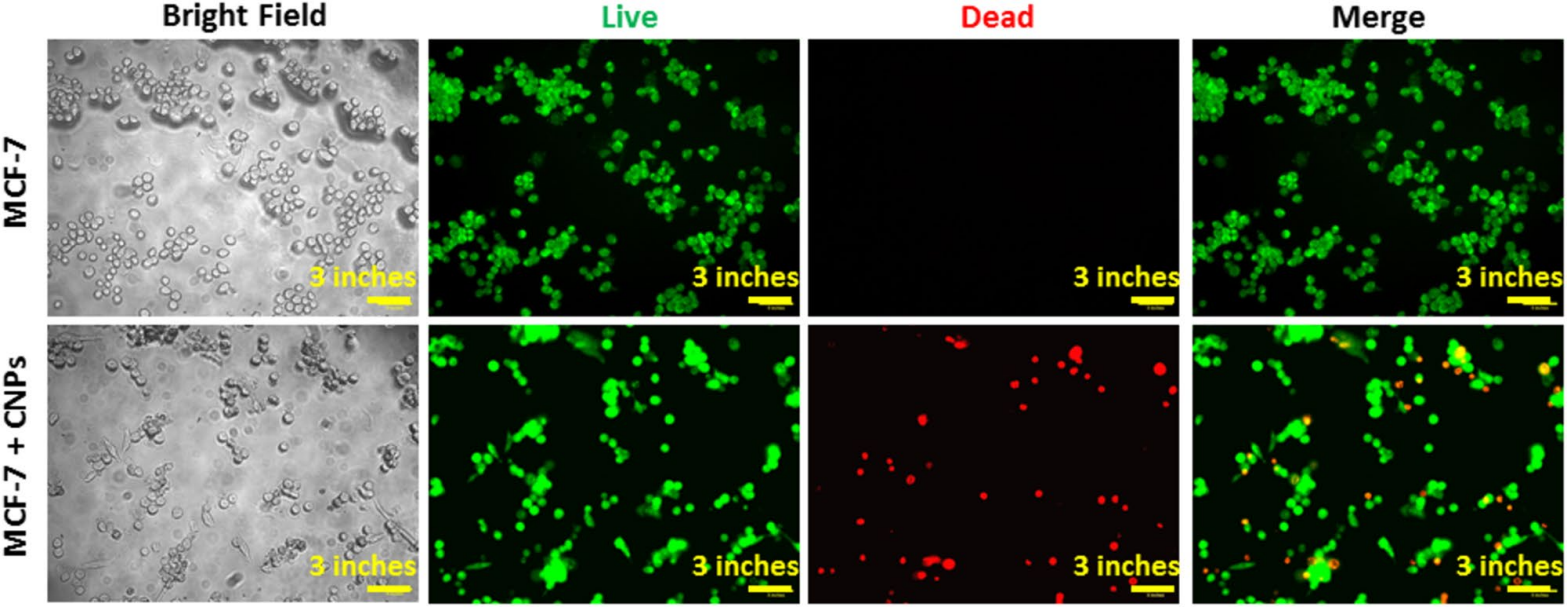
Cell viabilities were examined using Live & Dead cell assay kit Invitrogen. Images include the bright-field (BF) mode, Calcein, use E_x_/E_m_ ~ 485 nm/520 nm, green channel (live), For EthD-I, use Ex/Em ~ 530 nm/620 nm, red channel (dead). Confocal microscopic imaging of MCF-7 cells after incubation with 80 μg/ml of the CNPs.

The dye calcein AM is specifically capable of staining viable MCF-7 cells, resulting in green fluorescence. On the other hand, the dye Ethidium Homodimer-1 can only enter cells with compromised cell membranes, and it stains dead cells with a red fluorescence. The Live/Dead assay results, illustrated in Fig. 9, offer valuable insights into the effects of nanocrystalline CNPs on MCF-7 cancer cells. After incubation with these samples at a concentration of 80 µg/ml for 24 h, a significant increase is observed in the proportion of dead cells. This corroborates the findings of the MTT assay and confirms the cytotoxicity of the biosynthesized CNPs against the MCF-7 cell line. Microscopic images of untreated MCF-7 cells exclusively exhibit green staining, indicating that they are viable cells with intact membranes. Despite the concentration of the live/dead assay being below the IC50 value, MCF-7 cells treated with nanocrystalline CNPs show partial red staining, indicating damage to their cell membranes caused by the CNPs.

## Discussion

### Key properties of CNPs for biomedical applications

**Figure 10:**
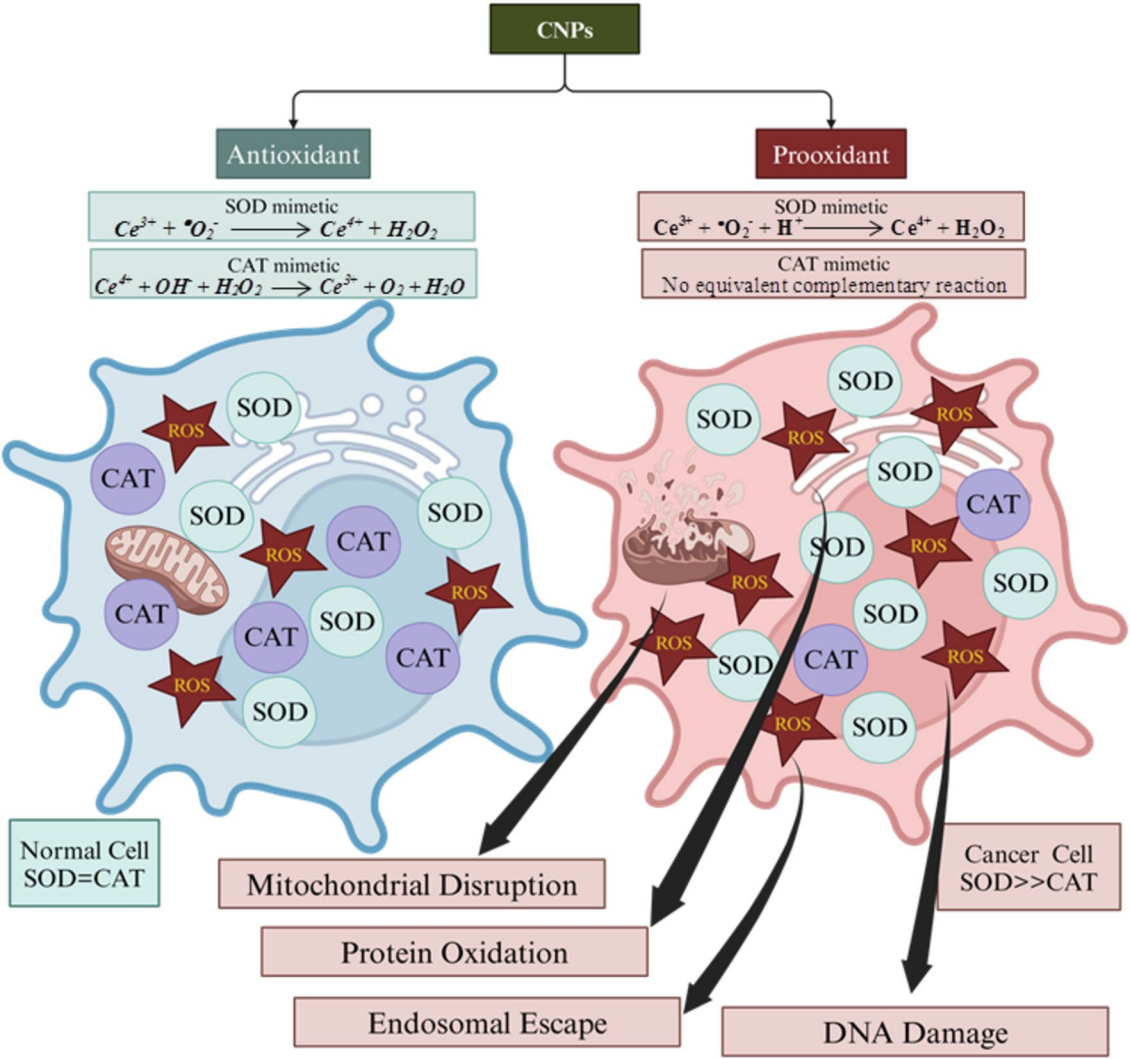
Mechanism of anticancer activity of CNPs.

CNPs are nanocrystalline particles derived from cerium (the most abundant rare earth element), which predominantly exist in the form of ceria with a unique face-centered cubic fluorite lattice structure. The high redox activity of CNPs is attributed to the rapid and convenient transition between Ce^3+^ and Ce^4+^ oxidation states, resulting in the generation of oxygen vacancies or defects in the lattice structure of CeO_2_ and CeO_2−×_^58^. Ceria possesses a high oxygen storage capacity and oxygen mobility within the lattice, making it widely applicable in biological processes related to oxidation/redox reactions^59^. The surface of CNPs, characterized by a higher surface-to-volume ratio compared to bulk material, exhibits softer atomic lattices. The smaller particle size and increased surface-to-volume ratio lead to the creation of more oxygen vacancies, which serve as active sites for reduction reactions^60–62^. While the catalytic mechanisms of simulated enzymes exhibit some precision, such as automatic recovery after redox reactions and substrate binding, there are still aspects that require further investigation^63–65^. The ability of CNPs to mimic the activity of multiple enzymes makes them highly convenient for biomedical applications that rely on redox activity, including anti-inflammation, antimicrobial properties, anticancer, angiogenesis, and more. Moreover, the excellent catalytic performance of CNPs enables their versatile application in photochemistry, electrochemistry, solid oxide batteries, organic pollutant degradation, high-performance catalysts, sensors, abrasive particles, coating materials, and various other industrial applications^66–68^. CNPs are recognized for their significant potential in biomedical applications, particularly as an effective anticancer agent. It offers cytoprotection to healthy cells by mitigating the harmful effects of ROS, while selectively inducing the formation of ROS to kill cancer cells. The mechanism underlying the anticancer activity of CNPs is depicted in Fig. 10^69^.

### Mechanism of anticancer activity of CNPs

Nanotechnology in cancer therapy, a relatively new approach for cancer treatment, relies on the nanocatalytic generation of ROS through Fenton or Fenton-like reactions^70^. These ROS possess the ability to destroy cancer cells by causing damage to biomolecules such as lipids, proteins, enzymes, and DNA, as well as organelles like mitochondria^71,72^. Recent research has highlighted the utility of ROS in exploiting the disparities between the physiological environment and the tumor microenvironment (TME). The TME exhibits abnormal characteristics including acidosis, hypoxia, inflammation, excessive hydrogen peroxide production, and vascular irregularities. While a balanced intracellular level of ROS is essential for the regulation of normal biological functions such as protein activation or inhibition, DNA mutagenesis, gene transcription activation, and antimicrobial effects, elevated levels of ROS, referred as oxidative stress, are associated with various disorders including cancer, cardiovascular disease, neurological diseases, inflammatory diseases, and diabetes^73^. The role of CNPs in modulating the production and maintenance of ROS levels is often considered in relation to key some of these species like superoxide, as well as their important regulators such as superoxide dismutase (SOD) and catalase (CAT)^74^.

The enzyme SOD plays a crucial role in protecting and repairing cells from damage caused by superoxide (^•^O_2_^−^) in the body. Therefore, an increase in SOD gene expression indicates the activation of the antioxidant defense mechanism. SOD also prevents the oxidative inactivation of nitric oxide (^•^NO), a reactive nitrogen species (RNS), thereby inhibiting the formation of peroxynitrite (ONOO^−^), another RNS^75^. Interestingly, the excessive production of SOD can be utilized in cancer therapy to generate ROS and the highly oxidizing agent H_2_O_2_. Since these reactions involve H_2_O_2_ as a reactant or product, they collectively contribute to the regulation of ROS for the benefit of healthy cells and the destruction of cancer cells. Thus, the role of CNPs in ROS regulation can be summarized by three concepts. Firstly cancer cells can be selectively killed through these reactions because their ROS levels are already elevated, allowing the ROS level to surpass the cytotoxic threshold. Secondly normal cells can maintain homeostasis even when the ROS level is temporarily raised above normal levels because it does not reach the cytotoxic threshold and subsequently returns to normal levels^76^. The Ce^3+^  ↔ Ce^4+^ redox switch and its associated ROS production represent an autoregenerative antioxidant mechanism that can be modulated by environmental pH levels. The microenvironment of cancer cells is typically acidic (pH ∼6.4, favoring Ce^4+^ as a prooxidant), while the physiological environment is slightly basic (pH ∼7.4, favoring Ce^3+^ as an antioxidant)^77^.

### Factors contributing to the anticancer activity of biosynthesized CNPs

The inhibition effect of biosynthesized CNPs against MCF-7 cancer cell lines could primarily be attributed to their small size, spherical morphology of substructures, maximum absorbance in the UV region, and prooxidant nature in the cancer cells' environment.

#### Morphology, shape, and size

The morphology, shape, and size of CNPs play a critical role in various aspects of cancer drugs, such as circulation time, biodistribution, cellular uptake, and targeting. Most nanoparticles designed for anticancer drugs are typically produced in a spherical morphology. Spherical nanoparticles are less affected by shear forces and exhibit stronger interactions with cell surfaces, thereby enhancing cellular uptake and therapeutic effectiveness^77^. Previous studies have shown that biosynthesized CNPs with nano-sized domains and a spherical morphology exhibit superior cytotoxicity against different cancer cell lines. However, agglomeration can lead to the formation of particle clusters, hindering cellular uptake and reducing the available surface area for interaction with cancer cells^78,79^. Therefore, achieving small size and desired morphology with minimal or no agglomeration is crucial for maximizing the anticancer efficacy of nanoparticles. In a study conducted by Panneerselvam et al., the relationship between calcination temperature and agglomeration was examined at various temperatures (300 °C, 400 °C, 500 °C, and 600 °C). Lower calcination temperatures (300 °C, 400 °C, and 500 °C) resulted in increased agglomeration, with mean particle sizes of 17 nm, 21 nm, and 24 nm, respectively. Although, biosynthesized CNPs calcined at 600°C exhibited a mean particle size of 31 nm in the mentioned study and a spherical morphology with less agglomeration and more uniform distribution^77^. In current study, we used *E. camaldulensis* as a biomodulator, capping, and oxidizing agent to synthesize CNPs with a calcination temperature of 400 °C. These biosynthesized CNPs exhibit spherical substructure morphology due to the TEM and SEM images. To determine the effect of agglomeration of these CNPs, an uptake study was performed on the MCF-7 cell line. The results have shown that CNPs uptaken successfully into these cancerous cells. These results indicated minimal or no agglomeration and desired shape, morphology, and size for anticancer studies.

#### Optical properties

The optical properties of CNPs provide valuable insights into their potential interactions with light and their applications in cancer treatment. As depicted in Fig. 3, biosynthesized CNPs exhibit maximum absorbance in the UV region. This characteristic allows the excited electrons to efficiently generate ROS by absorbing the most energetic part of the electromagnetic radiation. Due to its superior electronic excitation and band gap value of 3.43 eV, CNPS can produce a greater number of free radicals and increase intracellular ROS levels^78^. During the photo-excitation of the CNPs, an electron (e^−^) from the valence band (VB) is transferred to the conduction band (CB) when it absorbs incident energy equal to or greater than the bandgap energy. This process creates a hole (h^+^) in the VB, resulting in the formation of an exciton bound state of the e^−^ and h^+^ pair. Excitons are less stable but possess redox properties and initiate the ROS process. In the VB, the formed h^+^ oxidizes water molecules (H_2_O) and generates hydroxyl radicals (OH^•^) and hydrogen ions (H^+^). Meanwhile, in the CB, the excited e^−^ reduces oxygen (O_2_) molecules, producing superoxide radicals (^•^O^−^_2_), which combine with H^+^ to form HO^•^_2_ radicals. Furthermore, HO^•^_2_ can react with h^+^ ions to generate H_2_O_2_, which subsequently reacts with e^−^ to form OH^•^ radicals. Hence, CNPs trigger the production of intracellular ROS within the microenvironment. When the production of intracellular ROS exceeds the antioxidant defense mechanism of the cell, it leads to the formation of oxidative biomolecules, ultimately causing cell death^23,79–81^.

#### Prooxidative nature

The observed activity of biosynthesized CNPs in cancer cells may be attributed to its prooxidant nature within the cancerous cells' microenvironment. Cells rely on maintaining a balanced redox state, characterized by low levels of ROS, for proper functioning and signaling. Normal cells are equipped with antioxidant systems, including enzymes like SOD and CAT, to regulate ROS levels and maintain redox homeostasis. However, when there is an imbalance in redox equilibrium, the antioxidant mechanisms become ineffective, leading to excessive ROS generation, oxidative stress, and irreversible damage to proteins, lipids, and DNA^82^. In a neutral pH environment, CNPs exhibit its antioxidant properties in normal cells. At this state, CNPs with a normal redox level (Ce^3+^/Ce^4+^) function similarly to antioxidant enzymes like SOD and CAT, facilitating the decomposition of ROS and acting as a cytoprotective agent. However, in an acidic environment characteristic of cancer cells, CNPs act as a prooxidant due to their higher redox level (Ce^3+^/Ce^4+^). In this scenario, CNPs cause oxidative stress by generating a significant amount of ROS species, potentially triggering apoptosis in cancer cells (Fig. 10) ^83,84^. Therefore, the small size, spherical morphology, and advantageous optical properties of the synthesized CNPs collectively contribute to its prooxidant characteristics against the MCF-7 cancer cell line, leading to the accumulation of ROS and the induction of apoptosis. The current in-vitro cell-based studies demonstrate the potential anticancer efficacy of biosynthesized CNPs against MCF-7 breast cancer cell lines. However, further investigations, including ex-vivo and in-vivo studies, are necessary to fully understand the precise mechanism of action and evaluate the feasibility of using CNPs nano-formulation in clinical settings.

## Conclusion

In summary, the current study established that aqueous extracts of *E. camaldulensis* leaves were effectively employed as both a reducing and capping agent in the synthesis of CNPs. The first indication of the formation of CNPs was observed through a noticeable change in the color of the solution. FT-IR and EDX confirmed the elemental composition of CNPs and revealed the influence of biomolecules of plant extract during the synthesis process. The average size of the CNPs was determined to be 13.43 nm, and 39.25 nm using PXRD, and TEM, respectively. Biosynthesized CNPs exhibited unique structural and morphological characteristics with high surface area due to very small sphere-like Substructures as a result of the presence of biomolecules during the synthesis process. Furthermore, to make sure of the repeatability of this study's biosynthesis process, we repeated it three times and the obtained powders have been characterized using FT-IR, DR and XRD confirming the repeatability of the results. Cytotoxicity test on MCF-7 BC cell line was used to evaluate the anticancer activity of CNPs by the MTT assays. The findings indicate that the CNPs synthesized in this study have the ability to inhibit the growth of MCF-7 cancerous cells, suggesting their potential as a promising anti-cancer agent. To determine the apoptosis of the MCF-7 cell line in this study live dead assay was employed. Although the concentration of the live/dead assay utilized was lower than the IC50 value, the application of nanocrystalline CNPs on MCF-7 cells resulted in partial red staining, indicating the CNPs caused damage to the cell membranes. Confocal microscopy was used to confirm that the synthesized CNPs were taken up by MCF-7 cells and to investigate the pathway responsible for their cytotoxicity. The results showed a significant uptake of the nanoparticles by the MCF-7 cancerous cells. While synthesized CNPs via this eco-friendly approach show potential for inhibiting MCF-7 cell growth, further in-vitro and in-vivo research on biosynthesized CNPs such as assessment of the cytotoxicity against normal cells, evaluation of enzyme-mimic activity, and in-vivo studies is required to prove the anticancer activity of biosynthesized CNPs.

### Supplementary Information

Supplementary Information.

## Data Availability

Data availability The authors declare that the data supporting the findings of this study are available within the article.
