# Supplementary material for: In-vitro cytotoxicity of biosynthesized nanoceria using Eucalyptus camaldulensis leaves extract against MCF-7 breast cancer cell line
Source: Sci Rep. 2024 Jul 29;14:17465. doi: 10.1038/s41598-024-68272-3 (PMC11286930; doi:10.1038/s41598-024-68272-3)
Supplement: Supplementary file 1 — Supplementary Information. [file 41598_2024_68272_MOESM1_ESM.docx]

**Supporting Information**

In-vitro Cytotoxicity of Biosynthesized Nanoceria Using *Eucalyptus camaldulensis* Leaves Extract against MCF-7 Breast Cancer Cell Line

Fatemeh Abedi Tameh^1,2*^, Hamza Elsayed Ahmed Mohamed ^1^, Leila Aghababaee^3^, Mahmood Akbari^1*^, Shervin Alikhah Asl^1^, Mohammad Hasan Javadi^4^, Marique Aucamp^5^, Karen Jacqueline Cloete^1^, Janet Soleimannejad^2^ & Malik Maaza^1^

*^1^UNESCO-UNISA-iTLABS Africa Chair in Nanoscience and Nanotechnology, Muckleneuk Ridge, College of Graduate Studies, University of South Africa, P.O. Box 392, Pretoria 0003 South Africa*

*^2^School of Chemistry, College of Science, University of Tehran, P.O. Box 141556455, Tehran, Iran*

*^3^Bio Organic, Neuroscience Laboratory, Institute of Biochemistry and Biophysics (IBB), University of Tehran, 1417614335 Tehran, Iran*

*^4^Department of Chemistry, Sharif University of Technology, P.O. Box 11155‑9516, Tehran, Iran*

*^5^School of Pharmacy, University of the Western Cape, Robert Sobukwe Drive, Bellville, Cape Town, 7130, South Africa*

**Content**

**Supplementary Figure S1.** SEM images of biosynthesized CNPs….………………………………………**S2**

**Supplementary Figure S2.** EDX spectrum of CNPs.………………….…….………………………………**S3**

**Supplementary Figure S3.** TEM images of CNPs….……………….……………………………..………..**S4**

**SupplementaryFigure S4.** TEM images of CNPs….……………………..………………………..………..**S5**

| **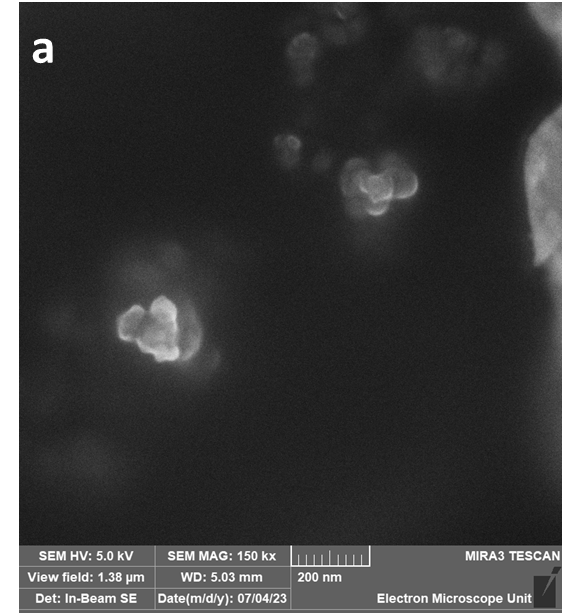** | **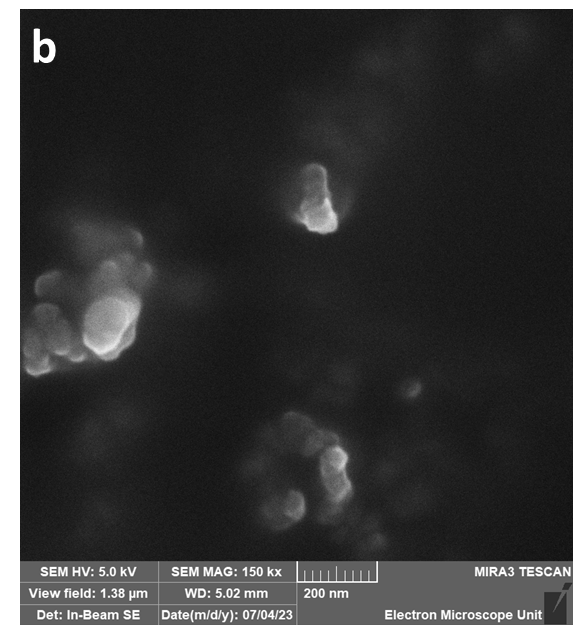** |
| --- | --- |
| **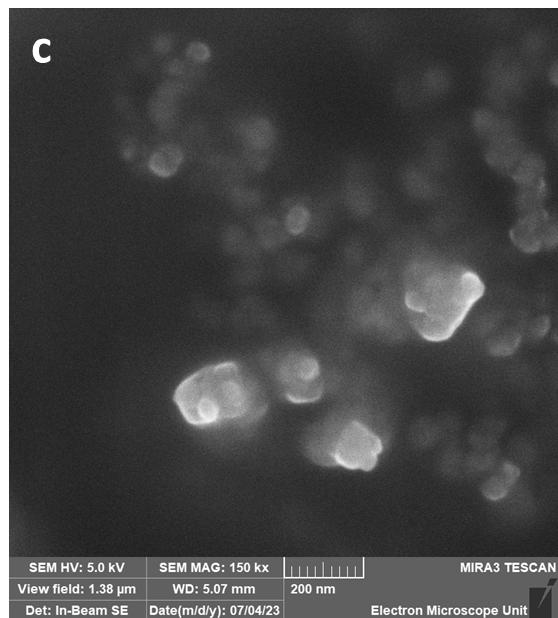** | |
| **Supplementary Figure S1.** SEM images of biosynthesized CNPs. | |
| 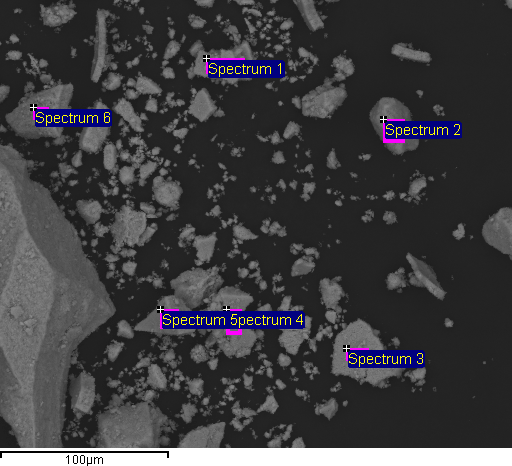 | |
| **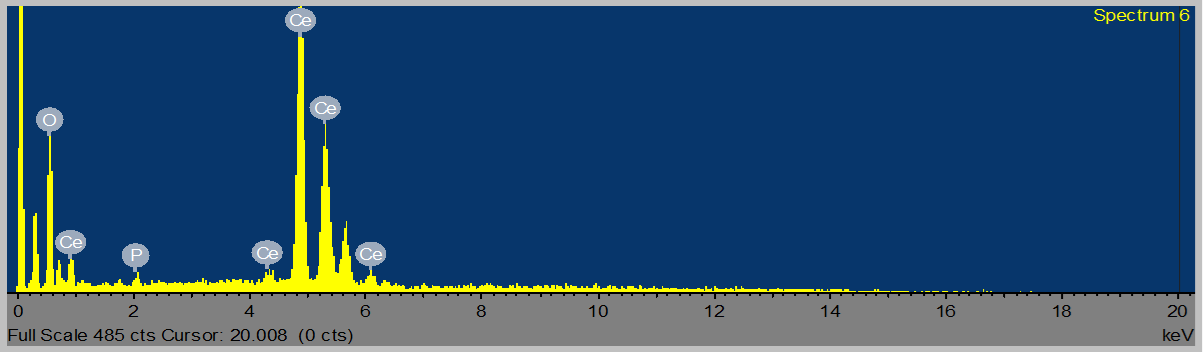** | |
| **Supplementary Figure S2.** EDX spectrum of CNPs. | |

| **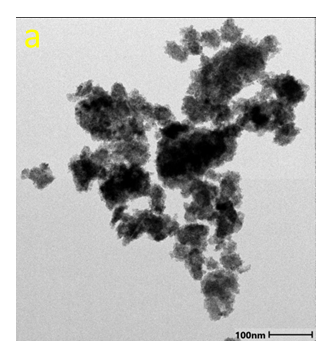** | **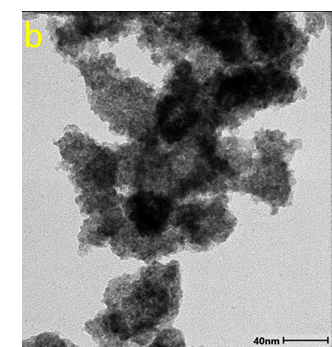** |
| --- | --- |
| **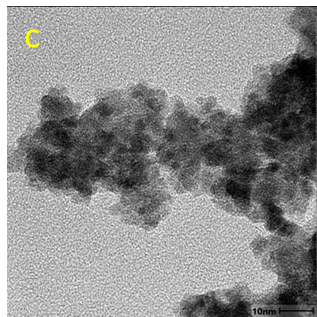** | **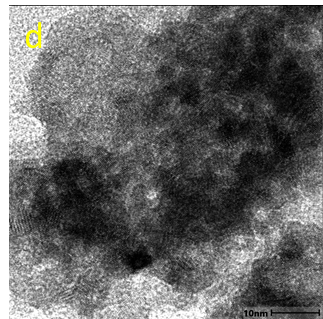** |
| **Supplementary Figure S3.** TEM images of CNPs. | |
| **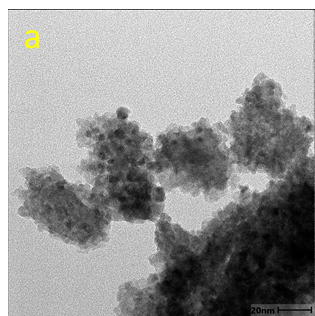** | **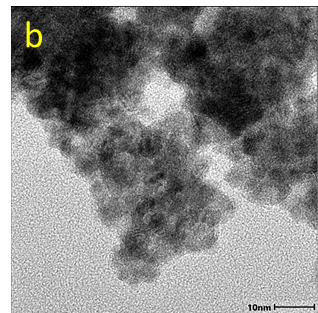** |
| **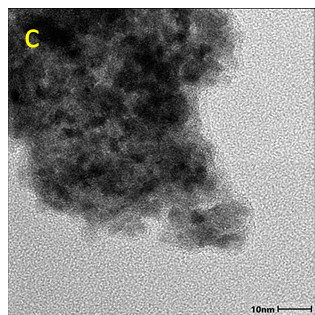** | **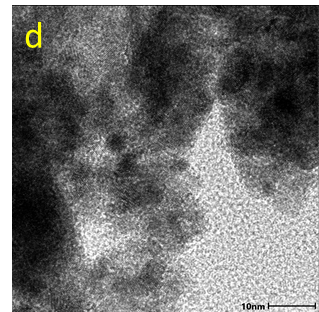** |
| **Supplementary Figure S4.** TEM images of CNPs. | |
